# Parental/grandparental education involvement and adolescents’ learning engagement: a moderated mediation model

**DOI:** 10.3389/fpsyg.2025.1564880

**Published:** 2025-08-07

**Authors:** Xiaoqing Xiang, Xiaofeng Huang, Mengyue Zhao, Jing Wang, Qian Shao

**Affiliations:** ^1^School of Psychology, Fujian Normal University, Fuzhou, China; ^2^Zhoukou Wentai Senior High School, Zhoukou, China; ^3^The Second Primary School of Cangwu Primary School Education Group, Lianyungang, China; ^4^College of Rehabilitation Science, Nanjing Normal University of Special Education, Nanjing, China

**Keywords:** grandparental education involvement, parental education involvement, personal growth initiative, learning engagement, moderated mediation model

## Abstract

Parental and grandparental involvement are associated with students’ educational success. However, few studies have explored the different effects of parental and grandparent education involvement on students’ academic performance. This study constructed a hypothetical model of parental/grandparental education involvement and adolescents’ learning engagement, with personal growth initiative as the mediating variable and self-education expectation as the moderating variable, to investigate how parental/grandparental education involvement influences learning engagement. A total of 822 adolescents from middle schools in China participated in the study. The results revealed that parental/grandparental education involvement significantly and positively affected adolescents’ learning engagement. In the context of parental education involvement, the most important factor in promoting learning engagement is emotional leisure. In the context of grandparental education involvement, the most important factor in promoting learning engagement is academic support. Furthermore, personal growth initiative mediated the relationship of parental and grandparental education involvement, respectively. The relationship between parental/grandparental educational involvement and learning engagement was moderated by self-education expectation. However, the interaction effect between self-education expectation and education involvement (emotional leisure, teaching rules, academic support, life care) to predict learning engagement is different between parents and grandparents. For the situation where parents directly raise their children, the interaction term (emotional leisure/teaching rules/academic support × self-education expectation) significantly predicted learning engagement, indicating that self-education expectation moderated the direct effect of emotional leisure, teaching rules, academic support on learning engagement. For the situation where grandparents providing caregiving for grandchildren, only the interaction term emotional leisure × self-education expectation significantly predicted learning engagement, indicating that self-education expectation moderated the direct effect of emotional leisure on learning engagement. The objective of this study was to provide further empirical evidence regarding the discrepancies in the mechanisms by which parental/grandparental education involvement affects adolescents’ learning engagement, and to offer further insights into the promotion of learning engagement among adolescents.

## 1 Introduction

Learning engagement has gained considerable attention as a vital determinant of students’ academic performance, as it represents an active and fulfilling learning state characterized by vigor, commitment, and concentration (Barberá-Tomás et al., 2022). Positive engagement can reliably predict current academic success (Moreira et al., 2013) and influence various aspects of students’ future lives. Actively engaged middle-school students exhibit lower dropout rates and fewer problem behaviors, positioning them favorably for future careers (Archambault et al., 2022). In China, parents highly emphasize their children’s education, as academic achievement is crucial for prestigious university admission and well-compensated employment (Moore, 2019). Academic success is considered the primary pathway to higher social status among junior-high-school students and is closely linked to future development (Guo et al., 2018). Studies have also indicated a declining tendency in junior-high-school students’ learning engagement between their first and third years (Zhou, 2021). Therefore, paying attention to this issue is of great importance for these students’ academic performance. Learning engagement is influenced by various factors, including individual characteristics, family dynamics, and school environment (Li et al., 2023). Among these, parental education involvement (PEI) significantly determines secondary school students’ academic achievement (Xiong et al., 2021).

With China’s socioeconomic development, many young people are occupied with work, leaving them with insufficient time and energy to care for their children. This has led to the common phenomenon of grandparents raising children. The World Population Prospects of 2019 suggest that the number of Chinese older adults aged 65 years or over will reach 366 million by 2050. Influenced by Confucianism, which emphasizes the collective family, many older adults in China shoulder extensive grandparenting responsibilities (Wang et al., 2022), and over 50% of Chinese grandparents provide childcare to grandchildren (Ko and Hank, 2014). Despite this prevalence, few studies have addressed the different effects of parental education involvement (defined as parent childcare, frequency of contact, provide financial support) and grandparental education involvement (defined as grandparent childcare, frequency of contact, the parents usually work in other places and provide financial support) on learning engagement. Moreover, research has shown discrepancies between parents and grandparents’ approaches to fostering children’s personal growth initiative (PGI; Sadruddin et al., 2019). Therefore, it is crucial to investigate, from the perspectives of parents and grandparents, the effects of educational involvement and PGI on learning engagement, as well as the underlying mechanisms involved.

### 1.1 PEI/GEI and learning engagement

PEI (parental education involvement) encompasses various behaviors that parents adopt to enhance students’ educational achievements based on diverse educational concepts and academic achievement expectations (Seginer, 2006). The positive impact of PEI on middle-school students’ learning engagement has been consistently emphasized in research (Chen et al., 2024). Active PEI correlates with enhanced academic outcomes, increased attendance rates, and overall student success. When parents engage in their children’s schooling, students demonstrate higher academic achievement, school engagement, and motivation (Barger et al., 2019). Studies among 9th and 10th graders in Jordan showed that PEI positively influenced students’ emotional engagement in school (Sapungan and Sapungan, 2014). This implies that students with more involved parents are more likely to find school enjoyable, have high self-esteem, and perceive school as a satisfying experience. Thus, parental support and guidance play a crucial role in positively affecting students’ learning.

Few studies have directly explored the relationship between GEI (grandparental education involvement) and learning engagement. However, current research suggests that grandparental involvement has a positive impact on children’s social skills and reduces the occurrence of internalizing and externalizing problem behaviors, which may subsequently influence adolescents’ learning engagement (Luo et al., 2020). Yorgason et al. (2011) investigated the association between grandparental involvement and positive outcomes for grandchildren. They found that, when grandparents are involved in their grandchildren’s daily lives, the grandchildren exhibit greater sociability and increased learning engagement. Therefore, we propose the following hypothesis:

*H1:* PEI and GEI are significant positive predictors of adolescent’s learning engagement.

### 1.2 PGI as a mediator

PGI encompasses cognitive and behavioral tendencies focused on active and intentional personal growth. This concept has been delineated into four relatively independent dimensions: readiness for change, planfulness, using resources, and intentional behaviors (Dengyong et al., 2014). According to the theory of self-determination, individuals generally have three basic needs: autonomy, ability, and relationships (Deci and Ryan, 2000). PGI is a manifestation of the need for autonomy as a vital intrinsic motivation that can significantly influence academic performance and emotional adaptation (Grolnick and Slowiaczek, 1994). Researchers have suggested that positive academic performance is challenging unless students autonomously engage in learning (Schnitzler et al., 2021). When students’ autonomous needs are met, they typically experience a sense of choice and self-recognition of their behavior (Deci and Ryan, 2008; Gagné and Deci, 2005). Consequently, they become more willing to participate and invest in learning activities. Studies have indicated that fulfilling students’ autonomy needs enhances their internal motivation, leading to more positive learning engagement behaviors (Reeve, 2012). Several studies have explored the relationship between PEI and students’ PGI, particularly among Chinese primary school students (Zhao et al., 2021). The findings reveal that the manner and extent of PEI positively impact students’ autonomy. Parent’s support and encouragement are crucial in cultivating students’ PGI, which closely correlates with their learning engagement (Li et al., 2023). In summary, PEI and GEI are advantageous for students’ PGI, and PGI is linked to improved learning engagement. This suggests that PGI may mediate the relationship between PEI/GEI and learning engagement. Thus, we propose the following hypothesis:

*H2:* PGI plays a mediating role in the link between PEI/GEI and learning engagement.

### 1.3 Self-education expectation as a moderator

Apart from PGI, an individual’s self-education expectation also modulates one’s implementation of learning activities. Students’ educational expectations are closely related to their academic performance (Pinquart and Ebeling, 2020). Students with higher self-education expectations work harder to learn to meet their expectations and achieve relatively better academic performance. One study proved that middle-school students’ self-education expectations has a positive impact on their academic development and can promote their degree of investment (Li et al., 2020; Shi et al., 2023). According to Bandura’s triadic interactive determinism, behavior, cognition, and the environment are interconnected and interact during the social learning process (Chai and Ye, 2024). Furthermore, the external environment influences individuals through inter-subjective factors. In this study, PEI and GEI were considered external environmental factors, while self-education expectation was considered an internal factor affecting individuals. Based on Bandura’s ternary interaction theory, junior-high-school students’ learning engagement is influenced not only by PEI, GEI, and self-education expectation, but also by their combined effect (Schmid and Garrels, 2021). Therefore, self-education expectation may act as a moderator in the pathway between PEI/GEI and learning engagement. Thus, we propose two hypotheses: self-education expectation significantly moderates the relationship between PEI and learning engagement (H3a), and self-education expectation significantly moderates the relationship between GEI and learning engagement (H3b).

### 1.4 The present study

**Figure 1:**
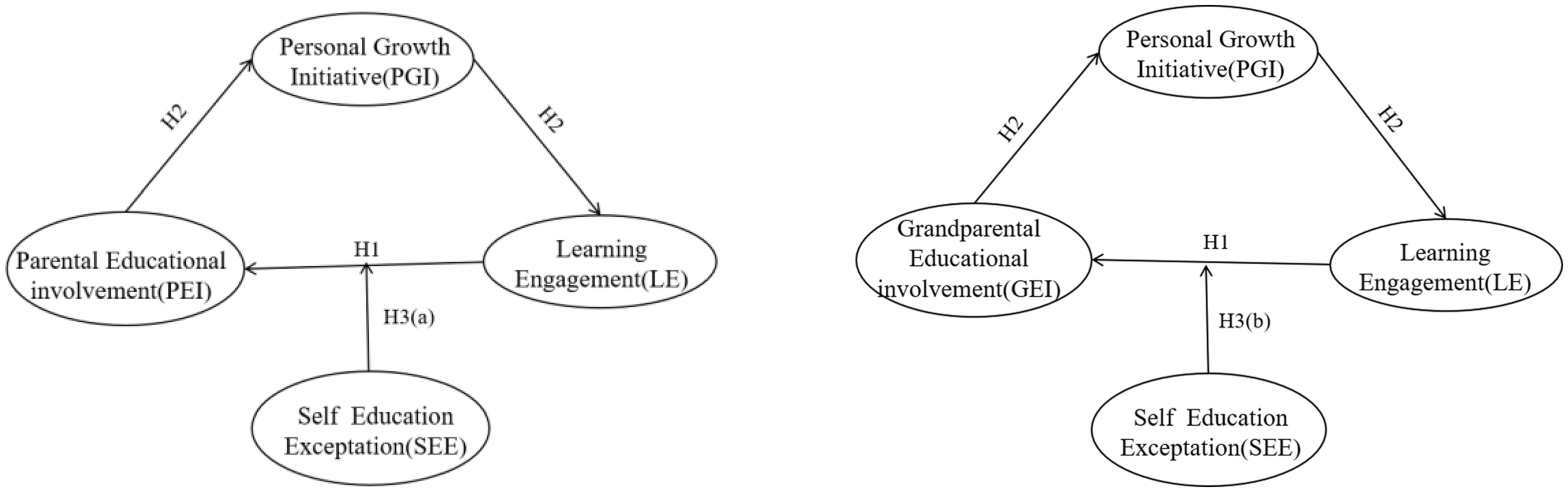
Hypothetical model. The two models depict the relationships between the main variables. The arrows indicate the mechanism of influence between the variables. The independent variable in the left model is assumed to be parental education involvement, whereas that in the right model is grandparental education involvement.

The present study examined the mediating and moderating factors in the association between PEI/GEI and learning engagement among adolescents in the Chinese context. It also explored the underlying mechanisms of this relationship by considering cognitive trait factors, including the mediating role of PGI and the moderating role of self-education expectation. By constructing two moderated mediation models (see Figure 1), this study contributes to the understanding of the mechanisms and differences underlying the influence of PEI and GEI on adolescents’ learning engagement. Additionally, it provides empirical support to guide adolescents’ learning engagement in a scientifically practical manner.

## 2 Methods

### 2.1 Sample and data collection

This study was approved by the Ethics Committee for Scientific Research at the authors’ institution. Data were collected from 850 junior-high-school students using a paper-based questionnaire. After excluding invalid responses, 822 responses were retained, resulting in a response rate of 96.7%. Among these valid responses, 380 were from male students and 442 were from female students, with ages ranging from 12 to 15 years (mean ± standard deviation: 12.16 ± 1.20). The participants were distributed across different grades, with 522 (63.5%) in Grade 1 of junior high school and 300 (36.4%) in Grade 2. Additionally, we categorized students based on their main caregivers, with 636 (77.4%) raised by parents and 186 (22.6%) raised by grandparents.

### 2.2 Measurements

#### 2.2.1 PEI/GEI

The study used the Parental/Grandparental Education Involvement Questionnaire (PIQ/GIQ), which was revised by Wu et al. (2018). The original questionnaire comprised 22 items across four dimensions: emotional leisure (11 items), and teaching rules (3 items), academic support (4 items), life care (4 items). Responses were scored on a 5-point scale, ranging from 1 (never) to 5 (always). Examples items are “Parents/Grandparents take care of your daily life,” “Parents/Grandparents exercise with me,” “Parents/Grandparents and I went on a trip,” and “Parents/Grandparents supervise my homework.” The questionnaire demonstrated good reliability and validity. Cronbach’s *α* coefficients of the PIQ in the present study were 0.73–0.92, and those of the GIQ were 0.71–0.89.

#### 2.2.2 Learning engagement

The Chinese version of the Utrecht Work Engagement Scale—Student, developed by Schaufeli et al. (2002) and revised by Fang et al. (2008), was used to measure adolescents’ engagement in learning. The scale comprises 17 items across three dimensions: vigor (six items; e.g., “I have much energy when I study”), commitment (six items; e.g., “I find studying valuable and meaningful”), and concentration (five items; e.g., “I am so concentrated that I forget everything when I study”). Participants responded to the items on a 7-point Likert scale, with scores ranging from 1 (never) to 7 (always). Cronbach’s alpha coefficient of the parent’s questionnaire in the present study were 0.89–0.90, and those of the grandparent’s questionnaire were 0.85–0.88.

#### 2.2.3 PGI

The Personal Growth Initiative Scale-II, developed by Robitschek et al. (2012), was used to assess PGI. This scale contains 16 items across four dimensions: ready for change (four items; e.g., “I know what aspects of myself need to change”), planning (four items; e.g., “I know how to make a realistic plan to change myself”), resource use (four items; e.g., “I know how to make a realistic plan to change myself”), and proactive behavior (four items; e.g. “I can take advantage of any opportunity to grow”). Items were scored on a 5-point scale ranging from 1 (strongly inconsistent) to 5 (strongly consistent). Cronbach’s alpha coefficient of the parent’s questionnaire in the present study were 0.78–0.85, and those of the grandparent’s questionnaire were 0.76–0.80.

#### 2.2.4 Self-education expectation

Self-education expectation was measured by asking participants about the highest level of education they wished to attain in the future. The response options and assigned values were: secondary vocational and below = 1, vocational college = 2, university = 3, and postgraduate and above = 4. Scores ranged from 1 to 4, with a higher score indicating higher expectations.

### 2.3 Data analysis

First, we conducted descriptive statistics and correlation analyses using IBM SPSS 26.0. Subsequently, we tested the mediation and moderated mediation models using the IBM SPSS macro PROCESS (Hayes, 2013), which has been widely used to test complex models, including mediated moderation and moderated mediation models. Models 4 and 5 in the PROCESS macro program^1^ were used to analyze the mediating role of PGI and the moderating role of self-education expectation between PEI/GEI and learning engagement. Additionally, previous research has revealed that learning engagement differs based on grade and previous achievement (Piñeiro et al., 2019; Zhou, 2021). Therefore, grade and previous achievement were included as control variables in this study.

## 3 Results

### 3.1 Descriptive and correlation analysis

The results in Tables 1, 2 illustrate the means (M), standard deviations (SD), and correlation coefficients of all the variables. Not all forms of parental involvement are positively related to academic achievement (Boonk et al., 2018). It is necessary to explore the different dimensions of parental/grandparental educational involvement. The four dimensions of PEI (*r* = 0.113–0.731, *p* < 0.01) and the four dimensions of GEI (*r* = 0.146–0.629, *p* < 0.05) demonstrated significant positive correlations with learning engagement, PGI, and self-education expectation.

**Table 1:** Descriptive statistics and bivariate correlations (parent).

Variable	*Min*	*Max*	*M*	*SD*	EL	TR	AS	LC	PGI	LE	SEE
EL	1	5	3.020	0.893	1						
TR	1	5	4.126	0.811	0.592^**^	1					
AS	1	5	3.351	0.939	0.731^**^	0.598^**^	1				
LC	1	5	4.076	0.814	0.543^**^	0.567^**^	0.553^**^	1			
PGI	1	5	3.397	0.811	0.446^**^	0.335^**^	0.359^**^	0.290^**^	1		
LE	1	7	4.466	1.351	0.427^**^	0.296^**^	0.350^**^	0.235^**^	0.561^**^	1	
SEE	1	4	3.448	0.656	0.204^**^	0.148^**^	0.143^**^	0.113^**^	0.310^**^	0.341^**^	1

EL, emotional leisure; TR, teaching rules; AS, academic support; LC, life care; PEI, parental education involvement; PGI, personal growth initiative; LE, learning engagement; SEE, self-education expectation.

**p < 0.01.

**Table 2:** Descriptive statistics and bivariate correlations (grandparent).

Variable	*Min*	*Max*	*M*	*SD*	EL	TR	AS	LC	PGI	LE	SEE
EL	1	5	2.842	0.816	1						
TR	1	5	4.032	0.889	0.452^**^	1					
AS	1	5	3.335	0.967	0.619^**^	0.596^**^	1				
LC	1	5	4.005	0.847	0.438^**^	0.462^**^	0.501^**^	1			
PGI	1	5	3.280	0.767	0.507^**^	0.376^**^	0.454^**^	0.442^**^	1		
LE	1	7	4.495	1.257	0.384^**^	0.336^**^	0.460^**^	0.384^**^	0.629^**^	1	
SEE	1	4	3.527	0.617	0.206^**^	0.146^*^	0.233^**^	0.189^**^	0.246^**^	0.320^**^	1

EL, emotional leisure; TR, teaching rules; AS, academic support; LC, life care; PEI, parental education involvement; GEI, grandparental education involvement; PGI Personal growth initiative; LE Learning engagement; SEE, self-education expectation.

*p < 0.05, **p < 0.01.

### 3.2 Multiple regression analyses

Separate multiple regression analyses were conducted using the four dimensions of PEI and GEI as independent variables. As shown in Tables 3, 4, we found that, after controlling for grade and prior academic achievement, the PEI results indicated that emotional leisure (*β* = 0.333, *p* < 0.001) had the most decisive influence, whereas the GEI results showed that academic support (*β* = 0.277, *p* < 0.01) was most influential.

**Table 3:** The results of multiple regression analyses (parent).

Model			*B*	*SE*	*β*	*t*	*p*	VIF
Constant			1.184	0.325		3.646	0.000	
Independent variable		Emotional leisure	0.504	0.080	0.333	6.323	0.000	2.452
		Teaching rules	0.033	0.078	0.020	0.422	0.673	1.911
		Academic support	0.095	0.076	0.066	1.253	0.211	2.479
		Life care	−0.001	0.073	−0.001	−0.020	0.984	1.727
Control variable	Grade	Grade1	0.431	0.097	0.154	4.437	0.000	1.069
		Grade2	0					
	Score ranking	Top	1.417	0.246	0.344	5.760	0.000	3.163
		Above the average	1.301	0.221	0.436	5.884	0.000	4.862
		Average	1.093	0.218	0.385	5.016	0.000	5.211
		Below the average	0.595	0.228	0.173	2.609	0.009	3.911
		Bottom	0					
*R^2^*					0.294			
*F*					28.869			
*p*					0.000			

**Table 4:** The results of multiple regression analyses (grandparent).

Model			*B*	*SE*	*β*	*t*	*p*	VIF
Constant			0.892	0.702		1.270	0.206	
Independent variable		Emotional leisure	0.100	0.121	0.065	0.823	0.412	1.738
		Teaching rules	0.055	0.109	0.039	0.499	0.619	1.682
		Academic support	0.361	0.115	0.277	3.137	0.002	2.196
		Life care	0.266	0.108	0.179	2.472	0.014	1.480
Control variable	Grade	Grade1	−0.023	0.475	−0.003	−0.048	0.962	1.053
		Grade2	0					
	Score ranking	Top	1.373	0.391	0.332	3.514	0.001	2.501
		Above the average	1.259	0.325	0.479	3.875	0.000	4.286
		Average	0.734	0.332	0.264	2.214	0.028	4.006
		Below the average	0.319	0.346	0.101	0.922	0.358	3.404
		Bottom	0					
*R^2^*					0.373			
*F*					11.647			
*p*					0.000			

### 3.3 Testing the mediation model

We employed Model 4 of PROCESS to examine the mediating effect of PGI between PEI/GEI and learning engagement. Percentile bootstrapping and bias-corrected percentile bootstrapping with 5,000 resamples were employed to construct 95% confidence intervals for the indirect effects. The four dimensions of parental/grandparental educational involvement represent different aspects of educational involvement, and they showed different correlations with learning engagement. Therefore, we will explore the relationship between educational involvement and learning engagement by dimension. After controlling for grade and previous achievement, four dimensions of parental education involvement significantly and positively predicted learning engagement in the absence of the mediator. Among them, the prediction power of emotional leisure is the strongest. Therefore, we mainly present the results of emotional leisure, as shown in Table 5.

**Table 5:** Mediation effect of personal growth initiative in emotional leisure and learning engagement (parent).

Outcome variable	Predictor variable	*B*	*β*	*SE*	*t*	*p*
LE	Grade	0.421	−0.152^***^	0.095	−4.420	0.000
	Achievement	0.320	0.250^***^	0.044	7.188	0.000
	EL	0.591	0.391^***^	0.052	11.425	0.000
PGI	Grade	−0.009	−0.006	0.060	−0.154	0.878
	Achievement	0.116	0.151^***^	0.028	4.154	0.000
	EL	0.384	0.422^***^	0.032	11.854	0.000
LE	Grade	−0.415	−0.150^***^	0.086	−4.839	0.000
	Achievement	0.239	0.187^***^	0.041	5.887	0.000
	EL	0.322	0.213^***^	0.051	6.263	0.000
	PGI	0.701	0.421^***^	0.057	12.252	0.000

LE, learning engagement; PGI, personal growth initiative; EL, emotional leisure.

***p < 0.01.

As observed in Tables 5, EL was positively correlated with PGI (*β* = 0.422, *p* < 0.001). When EL was controlled for, PGI was positively correlated with learning engagement (*β* = 0.421, *p* < 0.001). Moreover, when PGI was included, the association between EL and learning engagement remained significant (*β* = 0.213, *p* < 0.001). Finally, a bias-corrected percentile bootstrap method was used to test the mediation model. We generated 5,000 bootstrapping samples from the original data (*n* = 636 from parent data) by random sampling. The results (Table 6) indicate that the indirect effect of PGI was 0.269, and its 95% CI was [0.192, 0.350] in the parent data. This mediating effect accounted for 45.516% of the total effect of the association between ER and learning engagement. In other words, PGI partially mediated the association between EL and learning engagement.

**Table 6:** Bootstrap test for mediating effect (parent).

Model pathways		Effect	Boot LLCI	Boot ULCI	Ratio to total effect on LE
EL → PGI → LE	Indirect effect	0.269	0.192	0.350	45.516%
	Direct effect	0.322	0.221	0.423	
	Total effect	0.591	0.490	0.693	

EL, emotional leisure; PGI, personal growth initiative; LE, learning engagement.

Four dimensions of grandparental education involvement significantly and positively predicted learning engagement in the absence of the mediator, thus supporting H1. Among them, the prediction power of academic support (AS) is the strongest. Therefore, we mainly present the results of academic support, as shown in Table 7.

**Table 7:** Mediation effect of PGI in academic support and learning engagement (grandparent).

Outcome variable	Predictor variable	*B*	*β*	*SE*	*t*	*p*
LE	Grade	−0.052	−0.007	0.470	−0.110	0.912
	Achievement	0.406	0.349^***^	0.071	5.727	0.000
	AS	0.552	0.424^***^	0.079	6.966	0.000
PGI	Grade	0.070	0.015	0.305	0.229	0.819
	Achievement	0.143	0.202^**^	0.046	3.112	0.002
	AS	0.344	0.434^***^	0.051	6.704	0.000
LE	Grade	−0.105	−0.014	0.410	−0.256	0.798
	Achievement	0.297	0.255^***^	0.063	4.682	0.000
	AS	0.289	0.222^***^	0.077	3.750	0.000
	PGI	0.763	0.466^***^	0.100	7.657	0.000

LE, learning engagement; PGI, personal growth initiative; AS, academic support.

**p < 0.01, ***p < 0.001.

As observed in Table 7, AS was positively correlated with PGI (*β* = 0.434, *p* < 0.001). When AS was controlled for, PGI was positively correlated with learning engagement (*β* = 0.466, *p* < 0.001). Moreover, when PGI was included, the association between AS and learning engagement remained significant (*β* = 0.222, *p* < 0.01). Finally, a bias-corrected percentile bootstrap method was used to test the mediation model. We generated 5,000 bootstrapping samples from the original data (*n* = 186 from grandparent data) by random sampling. The results (Table 8) indicate that the indirect effect of PGI was 0.263, and its 95% CI was [0.164, 0.364] in the parent data. This mediating effect accounted for 47.645% of the total effect of the association between AS and learning engagement. In other words, PGI partially mediated the association between AS and learning engagement. Thus, H2 was supported.

**Table 8:** Bootstrap test for mediating effect (grandparent).

Model pathways		Effect	Boot LLCI	Boot ULCI	Ratio to total effect on LE
AS → PGI → LE	Indirect effect	0.263	0.164	0.364	47.645%
	Direct effect	0.289	0.137	0.441	
	Total effect	0.552	0.395	0.708	

AS, academic support; PGI, personal growth initiative; LE, learning engagement.

### 3.4 Testing the moderated mediation model

We used Model 5 of PROCESS to determine whether the direct effect was moderated by self-education expectation. We introduced an interaction effect between self-education expectation and education involvement (emotional leisure, teaching rules, academic support, life care) to predict learning engagement. The unstandardized model estimates for H3a and H3b are presented in Tables 9, 10, respectively. Due to space limitations, only the data results related to emotional leisure have been presented.

**Table 9:** Conditional process analysis in parent’s data (emotional leisure).

Regression equation	*B*	*SE*	*t*	LLCI	ULCI
Mediator variable model for predicting PGI					
Constant	3.035	0.136	22.308^***^	2.768	3.302
Grade	−0.009	0.060	−0.154	−0.126	0.108
Achievement	0.116	0.028	4.154^***^	0.061	0.170
EL	0.384	0.032	11.854^***^	0.320	0.447
Dependent variable model for predicting LE					
Constant	2.066	0.267	7.743^***^	1.542	2.590
Grade	−0.375	0.086	−4.348^***^	−0.544	−0.206
Achievement	0.208	0.042	4.916^***^	0.125	0.291
EL	0.317	0.051	6.209^***^	0.217	0.417
PGI	0.667	0.058	11.517^***^	0.553	0.781
SEE	0.119	0.072	1.644	−0.023	0.260
EL × SEE	−0.214	0.069	−3.112^**^	−0.349	−0.079

Conditional direct effect analysis at values of the moderator					
SEE	Effect	*SE*	*t*	LLCI	ULCI
Low (M − 1SD)	0.457	0.069	6.661^***^	0.323	0.592
Mean	0.317	0.051	6.209^***^	0.217	0.417
High (M + 1SD)	0.199	0.063	3.150^**^	0.075	0.323

PGI, personal growth initiative; EL, emotional leisure; LE, learning engagement; SEE, self-education expectation.

**p < 0.01, ***p < 0.001.

**Table 10:** Conditional process analysis in grandparent’s data (emotional leisure).

Regression equation	*B*	*SE*	*t*	LLCI	ULCI
Mediator variable model for predicting PGI					
Constant	2.720	0.333	8.177^***^	2.064	3.377
Grade	0.169	0.298	0.567	−0.418	0.756
Achievement	0.120	0.045	2.653^**^	0.031	0.209
EL	0.454	0.060	7.594^***^	0.336	0.572
Dependent variable model for predicting LE					
Constant	1.019	0.537	1.899	−0.040	2.078
Grade	−0.133	0.409	−0.325	−0.939	0.673
Achievement	0.252	0.064	3.931^***^	0.125	0.378
EL	0.102	0.094	1.078	−0.084	0.288
PGI	0.869	0.103	8.446^***^	0.666	1.071
SEE	0.172	0.115	1.494	−0.055	0.400
EL × SEE	−0.480	0.136	−3.533^**^	−0.748	−0.212

Conditional direct effect analysis at values of the moderator					
SEE	Effect	*SE*	*t*	LLCI	ULCI
Low (M − 1SD)	0.398	0.127	3.125^**^	0.220	0.796
Mean	0.102	0.094	1.078	0.045	0.502
High (M + 1SD)	−0.125	0.113	−1.107	−0.349	0.098

PGI, personal growth initiative; EL, emotional leisure; LE, learning engagement; SEE, self-education expectation.

*p < 0.05, **p < 0.01, ***p < 0.001.

**Figure 2:**
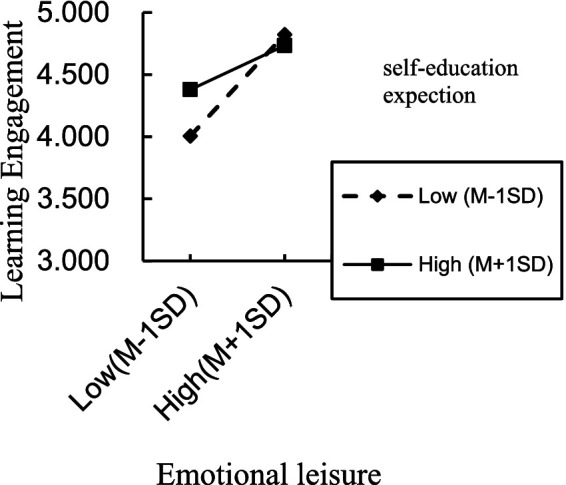
Interaction effect of self-education expectation and emotional leisure on learning engagement.

**Figure 3:**
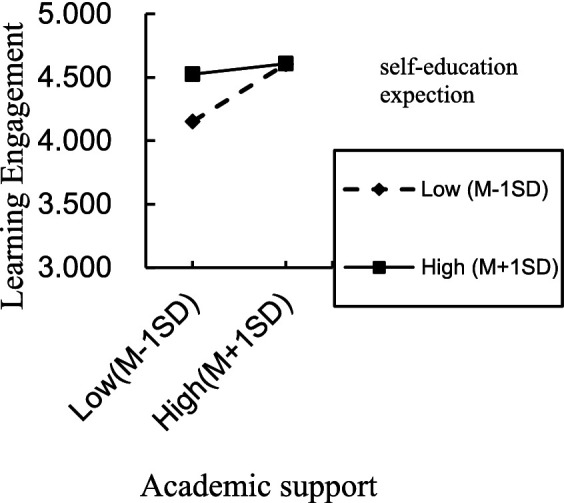
Interaction effect of self-education expectation and academic support on learning engagement.

**Figure 4:**
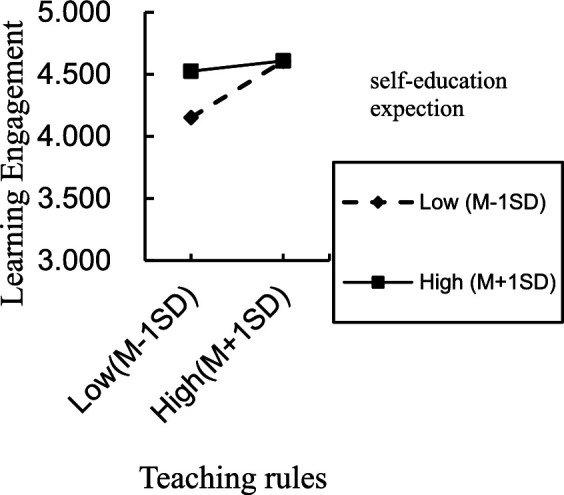
Interaction effect of self-education expectation and teaching rules on learning engagement.

For the situation where parents directly raise their children, the interaction term (emotional leisure/teaching rules/academic support × self-education expectation) significantly predicted learning engagement, indicating that self-education expectation moderated the direct effect of emotional leisure, teaching rules, academic support on learning engagement, thereby supporting H3a partially. The positive direct effect of emotional leisure, academic support on learning engagement was significant for individuals with low and high self-education expectation. However, the same positive direct effect of teaching rules was significant only for individuals with low self-education expectation. To better understand the moderating effect of self-education expectation, plots of the relationship between emotional leisure, academic support, teaching rules and learning engagement, at two levels of self-education expectation (1 SD below the mean and 1 SD above the mean) are depicted in Figures 2–4, respectively.

**Figure 5:**
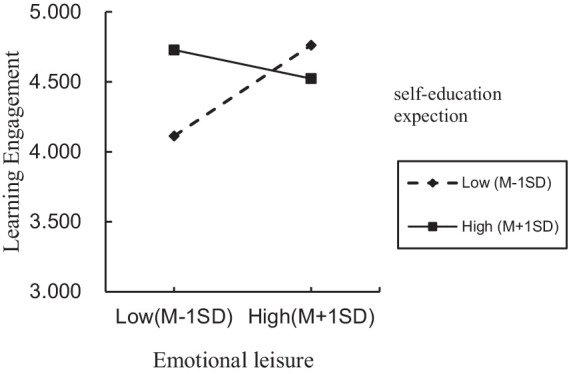
Interaction effect of self-education expectation and grandparental education involvement on learning engagement.

For the situation where grandparents providing caregiving for grandchildren, only the interaction term emotional leisure × self-education expectation significantly predicted learning engagement, indicating that self-education expectation moderated the direct effect of emotional leisure on learning engagement, thereby supporting H3a partially. Meanwhile, the positive direct effect of emotional leisure was significant only for individuals with low self-education expectation. To better understand the moderating effect of self-education expectation, plot of the relationship between emotional leisure and learning engagement, at two levels of self-education expectation (1 SD below the mean and 1 SD above the mean) is depicted in Figure 5.

As observed in Table 10 and Figure 5, for individuals with low self-education expectation, emotional leisure was positively associated with learning engagement (conditional effect = 0.398, *SE* = 0.127, 95% CI = [0.220, 0.796]). For individuals with high self-education expectation, the direct effect between emotional leisure and learning engagement was not significant (95% CI = [−0.349, 0.098]).

## 4 Discussion

This study explored the relationships between PEI/GEI and adolescents’ learning engagement in the Chinese cultural context, as well as the mechanisms underlying these relationships. The results indicated that PEI and GEI not only directly influenced learning engagement, but also exhibited an indirect effect mediated by PGI. For parental education involvement, among the four dimensions of emotional leisure, teaching rules, academic support, and life care, the correlation between emotional leisure and learning engagement is the greatest. For grandparental education involvement, among the four dimensions of emotional leisure, teaching rules, academic support, and life care, the correlation between academic support and learning engagement is the greatest. The interaction effect between self-education expectation and education involvement (emotional leisure, teaching rules, academic support, life care) to predict learning engagement are different between parent and grandparent. The findings contribute to a better understanding of how and when PEI and GEI are associated with learning engagement.

### 4.1 PEI/GEI and learning engagement

We observed that both PEI and GEI positively predicted adolescents’ learning engagement, which aligns with existing theoretical perspectives and empirical evidence. Positive parenting styles can directly influence adolescents’ psychological development (Bi et al., 2024). A positive association between PEI/GEI and heightened learning engagement levels ultimately predicts elevated academic achievement (Day and Dotterer, 2018; Hoover-Dempsey and Sandler, 1995; Skinner et al., 2009). This implies that individuals who experience greater PEI/GEI are more inclined to engage in learning activities. Drawing on social capital theory, family, as a significant social network, expands students’ social capital, providing more learning opportunities and resources. Meanwhile, four dimensions of parental education involvement significantly and positively predicted learning engagement in the absence of the mediator. In parents’ data, the prediction power of emotional leisure is the strongest. Compared with grandparents, parents are stricter with their children. Therefore, children need more leisure activities when they are with their parents. Positive emotional experiences from nature are of significance (Wang et al., 2025). Leisure activities positively predicted positive emotion (Zhang and Zheng, 2017) which helpful for learning engagement. In grandparents’ data, the prediction power of academic support is the strongest. Grandparents can, to a certain extent, make up for the lack of educational support caused by the absence of parents by means such as supervising homework and helping solve learning difficulties, thus enabling children to devote themselves to their studies.

### 4.2 Mediating role of PGI

Our study not only revealed that PEI, GEI, and PGI positively predict learning engagement, but also unveiled the mediating role of PGI in the relationships between PEI/GEI and learning engagement. This finding aligns with previous studies supporting the idea that students who experience PEI/GEI are more likely to value their learning, thus promoting autonomous motivation (Schmid and Garrels, 2021). According to Deci and Ryan’s self-determination theory (Vansteenkiste et al., 2020), individuals have three basic psychological needs: autonomy, relatedness, and competence. PEI and GEI play a crucial role in shaping children’s future development, fostering autonomy, and motivating active participation in self-regulated learning (Ahmed et al., 2024). When adolescents’ basic needs are satisfied, they are more motivated to engage in their growth process and have higher expectations for their future academic life (Leversen et al., 2012). Parents and grandparents who are positively involved in offering academic assistance or life care satisfy students’ psychological needs and foster their autonomous motivation for learning. As a vital component of an individual’s inner psychological resources, PGI plays a mediating role in the connections between PEI/GEI and adolescents’ learning engagement. A supportive family atmosphere fosters stronger autonomous motivation, and adolescents will have higher expectations for learning when they are effectively assisted in developing positive character traits such as PGI (Benneker et al., 2023). Furthermore, adolescents who grow up in such environments often have more positive experiences, experience better psychological wellbeing, and exhibit greater life satisfaction (Wang et al., 2018). Consequently, they are more likely to enjoy their academic lives, which in turn influences their learning engagement.

### 4.3 Moderating effect of self-education expectation

Our study revealed that adolescents’ self-education expectation moderated the relationships between PEI/GEI and learning engagement. In the data of the parents, the interaction term (emotional leisure/teaching rules/academic support × self-education expectation) significantly predicted learning engagement, indicating that self-education expectation moderated the direct effect of emotional leisure, teaching rules, academic support on learning engagement. In the data of grandparents, only the interaction term emotional leisure × self-education expectation significantly predicted learning engagement, indicating that self-education expectation moderated the direct effect of emotional leisure on learning engagement.

Specifically, these relationships were stronger among adolescents with lower self-education expectations than among those with higher expectations. According to existing research, adolescents with higher academic achievement expectations are more likely to prioritize personal effort and autonomous motivation (Shengyao et al., 2024). Conversely, when adolescents lack aspirations for improved academic achievement, PEI/GEI can instill a sense of responsibility and motivation, driven by the desire not to disappoint their parents/grandparents, thereby empowering them to tackle challenging learning tasks more effectively. In this context, PEI/GEI serves as a source of stability and reassurance, assisting adolescents in navigating diverse obstacles. Such experiences can enhance adolescents’ awareness of the support they receive from others, ultimately promoting their learning engagement (Luo et al., 2020).

However, the moderating mechanisms of self-education expectation may differ depending on adolescents’ perceived PEI/GEI. In the data of the parents, for individuals with low self-education expectation and high self-education expectation, the positive direct effects of both emotional leisure and academic support on learning engagement were significant. However, the positive direct effect of teaching rules on learning engagement was not significant for individuals with high self-education expectation. This means that, for individuals with high self-education expectations, teaching rules did not affect learning engagement. In the data of the grandparents, self-education expectation only moderated the direct effect of emotional leisure on learning engagement. For individuals with low self-education expectation, emotional leisure was positively associated with learning engagement. For individuals with high self-education expectation, the direct effect between emotional leisure and learning engagement was not significant. In China, the ways and values of parents and grandparents differ significantly (Lu et al., 2022). Parents were more influenced by modernization and urbanization, with a greater emphasis on academic performance, personal achievement, and social status. Thus, they may be more inclined to use modern educational resources and methods, such as cram schools and online education, in the hope that their children will stand out from the fierce competition (Chen et al., 2021). According to existing research, children’s learning engagement is positively related to both paternal and maternal involvement (Li et al., 2023). However, grandparents tend to pay more attention to the inheritance of traditional values (Zhang and Wu, 2021) or children’s health outcomes (Pulgaron et al., 2016). Thus, grandparents’ influence might differ owing to generational gaps and differing perspectives on education (Grolnick and Pomerantz, 2022). While grandparents may still play a role, their influence may be indirect or less pronounced than that of parents (Dunifon and Bajracharya, 2012).

### 4.4 Research significance and limitations

This study has important theoretical and practical implications. First, it aligns with the theory of positive adolescent development, which emphasizes how adolescents’ external environments interact with their inner strengths to shape their learning engagement. This offers effective strategies for fostering adolescents’ learning behaviors. Second, there is an urgent need to prioritize the promotion of autonomous motivation and initiative among adolescents. This can be achieved by clarifying their learning goals and fostering personal growth dynamics. These qualities play a pivotal role in adolescents’ learning behaviors and growth processes, equipping them with resilience to overcome challenges and fulfill their potential.

Regarding practical implications, this study considers the discrepancy mechanisms in which PEI/GEI affects students’ learning engagement. The situation where parents raise their children themselves, along with taking them to educational places like science museums, engaging in leisure activities such as traveling or exercising, can help enhance children’s learning engagement. When parents work in other places and the children are raised by their grandparents, the grandparents providing necessary academic support for the children can help increase their learning engagement. However, for the elderly with lower educational levels, this is very difficult to achieve. Meanwhile, the results of the moderation effect revealed that parents play a more important role in contributing to children’s learning.

Despite the valuable insights provided by this study, it has some limitations. First, the study’s scope was confined to one secondary school in China, potentially introducing regional bias and limiting the generalizability of the results. To enhance the reliability and applicability of our findings, future research should expand the sample size and consider diverse cultural contexts. Second, longitudinal studies and follow-up surveys could offer a more dynamic and holistic comprehension of PEI and adolescents’ learning engagement; thus, longitudinal studies should be conducted in the future.

## 5 Conclusion

Based on an analysis of the research data, a few conclusions were drawn in this study. PEI and GEI in middle-school students positively predict learning engagement. In a family environment that provides greater involvement and support, middle-school students tend to engage in more learning activities. In the context of parental education involvement, the most important factor in promoting learning engagement is emotional leisure. In the context of grandparental education involvement, the most important factor in promoting learning engagement is academic support. Meanwhile, individuals with a higher level of PGI are more willing to engage in learning. Additionally, in the context of parental education involvement, self-education expectation moderated the direct effect of emotional leisure, teaching rules, academic support on learning engagement. Meanwhile, the effect of emotional leisure, academic support on children’s learning engagement was stronger among individuals with lower self-education expectation. The effect of teaching rules on children’s learning engagement was significant only among individuals with low self-education expectation. In the context of grandparental education involvement, self-education expectation only moderated the direct effect of emotional leisure on learning engagement. Meanwhile, the effect of emotional leisure on children’s learning engagement was significant only among individuals with low self-education expectation.

## Data Availability

The raw data supporting the conclusions of this article will be made available by the authors without undue reservation.

## Footnotes

^1^ http://www.afhayes.com
